# Rate of chronic otitis media operations and cholesteatoma surgeries in South Korea: a nationwide population-based study (2006–2018)

**DOI:** 10.1038/s41598-020-67799-5

**Published:** 2020-07-09

**Authors:** Gi Jung Im, Kyung do Han, Kyung Ho Park, Chang Hyun Cho, Hyunsook Jang, Jun Ho Lee, Seung Hwan Lee

**Affiliations:** 1 Department of Otolaryngology-Head and Neck Surgery, Korea University College of Medicine, Goryeodae-ro, Seongbuk-gu, Seoul, 02841 South Korea; 2 Department of Statistics and Actuarial Science, Soongsil University, Seoul, South Korea; 3 Department of Otolaryngology-Head and Neck Surgery, Catholic University College of Medicine, Seoul, South Korea; 4 Department of Otolaryngology-Head and Neck Surgery, Gachon University College of Medicine, Incheon, South Korea; 5 Division of Speech Pathology and Audiology, Hallym University College of Natural Sciences, Chuncheon, South Korea; 6 Department of Otolaryngology-Head and Neck Surgery, Seoul University College of Medicine, Seoul, South Korea; 7 Department of Otolaryngology-Head and Neck Surgery, Hanyang University College of Medicine, Seoul, Korea

**Keywords:** Computational biology and bioinformatics, Diseases, Health care

## Abstract

The aim of this study was to estimate the total number and rate of chronic otitis media (COM) operations and cholesteatoma surgeries in South Korea, using a nationwide survey which analysed a 13-year trend (2006–2018). This study also analysed the trend of COM operations and cholesteatoma surgeries according to year, sex, and age using a nationwide population-based database, and the 13-year trend was analysed according to age groups. This study used nationwide data from the National Health Information Database (NHID), which is a government-affiliated agency under the Korean Ministry of Health and Welfare that supervises all medical activities in Korea. Retrospective medical data of patients of all ages were extracted from the NHID from January 2006 to December 2018 (NHIS-2018). This study was conducted by the Research Committee of the Korean Society of Otorhinolaryngology-Head and Neck Surgery, and the Korean Audiological Society reviewed and confirmed the study. There was a 1.5 fold increase in COM operation rates in 2018, compared to 2007 figures. The annual total number of COM operations was 5,935 in 2007, 8,999 in 2012 (peak), and 8,870 in 2018 (17 in 100,000). Meanwhile, the total annual number of cholesteatoma surgeries decreased from 3,502 in 2006 to 3,199 in 2018 (6 in 100,000). The rate of COM operations was higher (1.27 fold) in the female population than in the males in 2018. However, cholesteatoma surgery rates were higher (1.2 fold) in the male population than in the females in 2018. According to the 2018 data, COM operations were most commonly performed in patients in their 50s. COM operation rates increased rapidly in patients aged 51–80. In other age groups however, rates were constant or showed a decrease in figures, especially in the 40s age group (1st rank in 2006 to 3rd rank in 2018). According to the 2018 data, cholesteatoma surgery was most commonly performed in patients in their 50s. Cholesteatoma surgery rates increased dramatically from 2006 to 2018 in patients aged 0–10 years due to congenital cholesteatoma. Cholesteatoma surgery rates also increased in patients aged 61–80 years due to ageing population. Cholesteatoma surgery rates decreased in patients aged 41–50 years, ranking 1st in 2006 and 4th in 2018. In conclusion, the annual rate of COM operations was 0.017%, and no longer increases, but stabilizes/decreased after a peak point in the advanced country. The mean rate of cholesteatoma surgery was 0.006%, and decreased annually. There was female dominance in COM operations, but male dominance in cholesteatoma surgery. Major age groups of patients who underwent COM/cholesteatoma surgery were the 50s and 60s, and congenital cholesteatoma (0–10 years) accounted for about 20% of all cholesteatoma surgery.

## Introduction

Recurrent infections of the middle ear and mastoid cavity result in a permanent perforation of the tympanic membrane. Chronic otitis media (COM) means chronic or recurrent infection of ears with chronic tympanic perforation. Chronic infection of the mastoid cavity induces severe granulation and related infection, and finally contracted or sclerotic mastoid cavity. COM affects an estimated 330 million people worldwide, and about 60% of these patients have significant hearing loss^1–4^.

According to the WHO global burden of diseases and management options for chronic suppurative otitis media (CSOM) in the year 2004, the prevalence of CSOM was classified as highest (> 4%), high (2–4%), low (1–2%), and lowest (< 1%)^2^. The prevalence of CSOM was quite different by region and country, from 0.3 to 14%. The worldwide population of CSOM is 65 to 330 million persons, and 39 to 200 million (60%) of those individuals have clinically significant hearing impairment^3,5^.

In South Korea, several prevalence studies of CSOM have been conducted. In the population > 4 years of age (n = 25,147, 2009–2012), the prevalence of CSOM was 3.13%, the prevalence of obvious cholesteatoma was 0.34%, and the prevalence of CSOM increased with age (***P < 0.001) and had a female predominance (*P = 0.014)^6^. Of the 16,063 participants aged above 20 years (2010–2012), the weighted prevalence of COM was 3.8% and had a female predominance (*P = 0.0287)^7^.

In the United States, CSOM occurs at a rate of less than 1%, whereas in many developing countries higher rates of greater than 4% are observed^8^. Studies from Thailand, Vietnam, Korea, and Malaysia showed a prevalence of CSOM ranging from 0.9 to 4.7%^8^. A recent study in India had a higher prevalence of 7.8%^8^. Prevalence of CSOM is related to many factors including crowded habitation, access to medical care including vaccinations, antibiotics use, exposure to smoking, bottle feeding, and poor nutrition/hygiene^8^.

From various studies, the incidence rate of cholesteatoma was 3–15 per 100,000 person-years. The incidence rate reported in Denmark was 6.8 per 100,000 person-years^9^, and the mean annual incidence was 9.2 per 100,000 inhabitants in Finland^10^. Another nationwide study in Denmark described the incidence rate of surgically treated middle ear cholesteatoma in 3,874 Danish children (0–15 years) from 1977 to 2010; the incidence rate increased from 8 to 15 per 100,000 person-years from 1977 to 2002 and the rate decreased from 15 to 10 per 100,000 person-years from 2002 to 2010^11^. The annual incidence of cholesteatoma, including cases treated without surgery, was 6.8–10 in a population of 100,000 in Fukuoka city, Japan^12^. In a radiological study, the annual incidence of cholesteatoma was reported as 3 per 100,000 in children and 9.2 per 100,000 in adults with a sex ratio of 1.4:1, indicating male predominance^13^.

The aim of COM treatment is to create a dry, safe ear, and to preserve or restore hearing as much as possible. Tympanomastoidectomy directly affects both the middle ear and the mastoid cavity, because COM involves both cavities. Classification of tympanomastoidectomy includes (1) intact canal wall mastoidectomy (ICW); closed-cavity tympanomastoidectomy; canal wall-up technique, and (2) open-cavity mastoidectomy (OC); canal wall-down technique. Indications for mastoidectomy include history of profuse otorrhea with tympanic membrane perforations, cholesteatomas or tumors extending into the mastoid bone, previous tympanoplasty failure, and chronic ear infection which cannot be corrected. The success rates for sealing a tympanic perforation with a graft can be as high as 90–95%^1^. Hearing deficits may be corrected in about 50–70% of operated ears^1^.

South Korea has prominent advantages when conducting a nationwide study on hearing loss (HL) or otologic disease, and on rates of surgery or procedures. The reasons include (1) the territory is relatively small, (2) transportation or accessibility to medical services is excellent, and (3) the entire national population is registered with the Korean National Health Insurance Service (KNHIS), with all medical data being well organised in their respective National Health Information Database (NHID)^14–16^. Thus, the analysis of nationwide data on otologic surgery can provide the true rates and trends of middle ear surgery in South Korea.

The aim of this study was to estimate the total number and rates of COM operations and cholesteatoma surgeries in South Korea determined using a nationwide survey to analyse a 13-year trend (2006–2018). The study also analysed the trend of COM operations and cholesteatoma surgeries according to year, sex, and age by using a nationwide population-based database, and the 13-year trend was analysed according to age groups.

## Results

### The state of society in South Korea from 2006 to 2018

**Figure 1:**
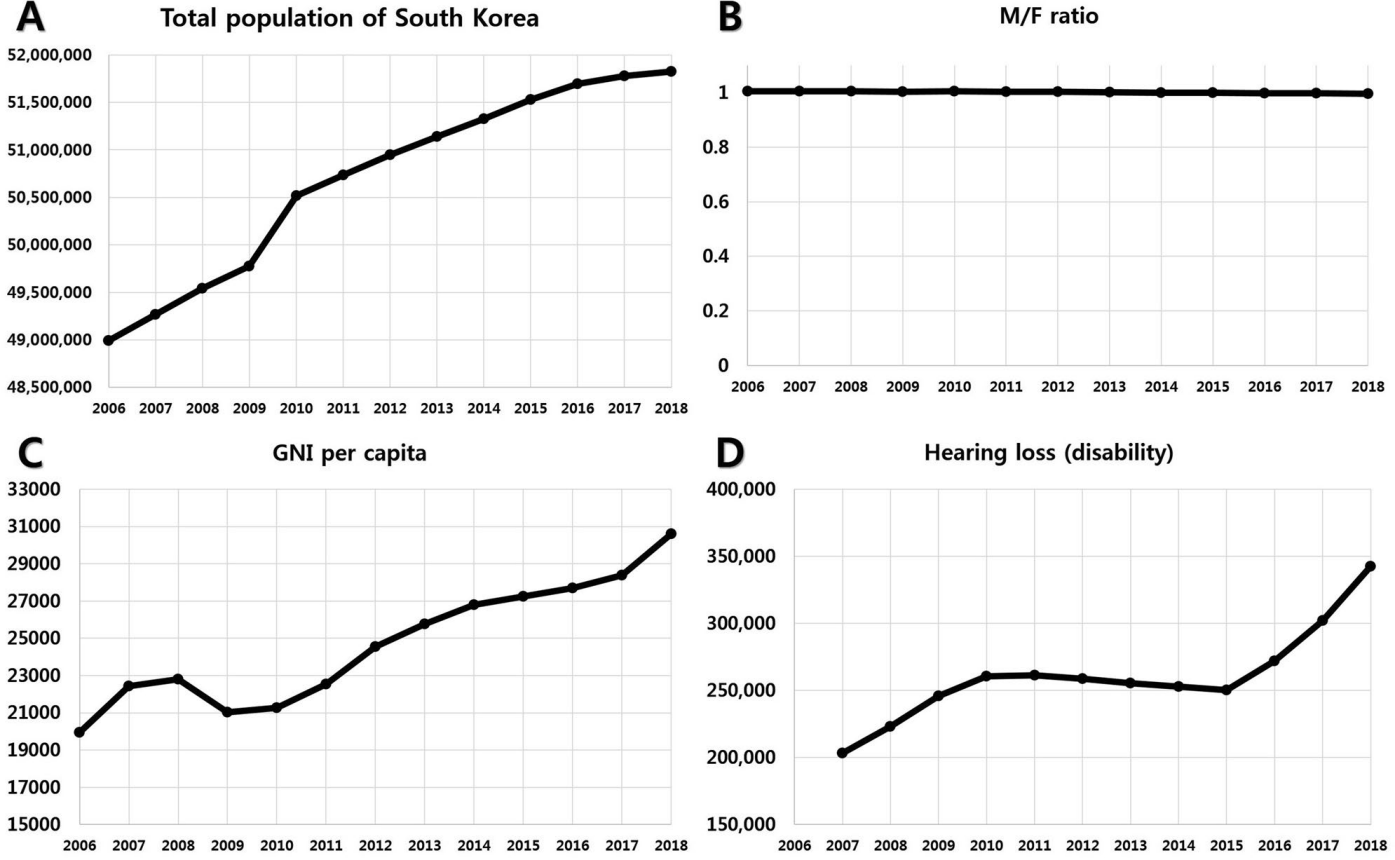
South Korea from 2006 to 2018 including the total population of South Korea, male/female (M/F) ratio, gross national income (GNI) per capita, and population of hearing disability which shows severe-profound hearing loss (HL) over bilateral 60 dB HL. Basic national data showed that the situation in South Korea is stable, and suitable for nationwide big data analysis. (**A**) The total population of South Korea continues to increase from 48.9 million to 51.8 million (2006–2018). (**B**) The M/F ratio of South Korea was almost same as 1:1 from 1.005 to 0.995 (2006–2018), and the female population increased as time went on. (**C**) GNI per capita of South Korea. From 2006 to 2018, GNI per capita continues to increase from 19,950 to 30,600. Annual increase of GNI was about 2.9%, and ranked 28th in the world in 2018. (**D**) The population with hearing disability which showed severe-profound hearing loss over bilateral 60 dB HL. Total number of people with hearing disability in 2007, which was the onset of data collection, was 203,324 and it increased to 250,334 by 2015. The trend of hearing disability formed a gradual decrease from 2010 to 2015. However, hearing disability increased to 342,582 from 2015 to 2018 because of the start of the hearing aid funding programme of the South Korean Government.

From 2006 to 2018, the total population of South Korea continued to increase from 48.9 million to 51.8 million. An approximate 0.44% annual increase was observed in the total population of South Korea, and formed a plateau, steady state that neither increases nor decreases (Fig. 1A). Sex ratio, male/female (M/F) ratio was almost the same throughout (1:1 from 1.005 to 0.995; Fig. 1B). From 2006 to 2018, gross national income (GNI) per capita continued to increase from $19,950 to $30,600. The annual increase of GNI per capita was about 3.4% and ranked 28th in the world in 2018. (Fig. 1C). In South Korea, hearing disability is defined as severe-profound HL over bilateral 60 dB HL, which can get a national support for hearing aid. The total number of individuals with hearing disability in 2007 was 203,324, and it increased to 250,334 in 2015. The trend of hearing disability formed a gradual decrease from 2010 to 2015. However, the number of people with hearing disability increased to 342,582 from 2015–2018 temporarily because of the start of the hearing aid funding programme of the South Korean Government (Fig. 1D).

### The total number of COM operations in South Korea (2006–2018)

**Figure 2:**
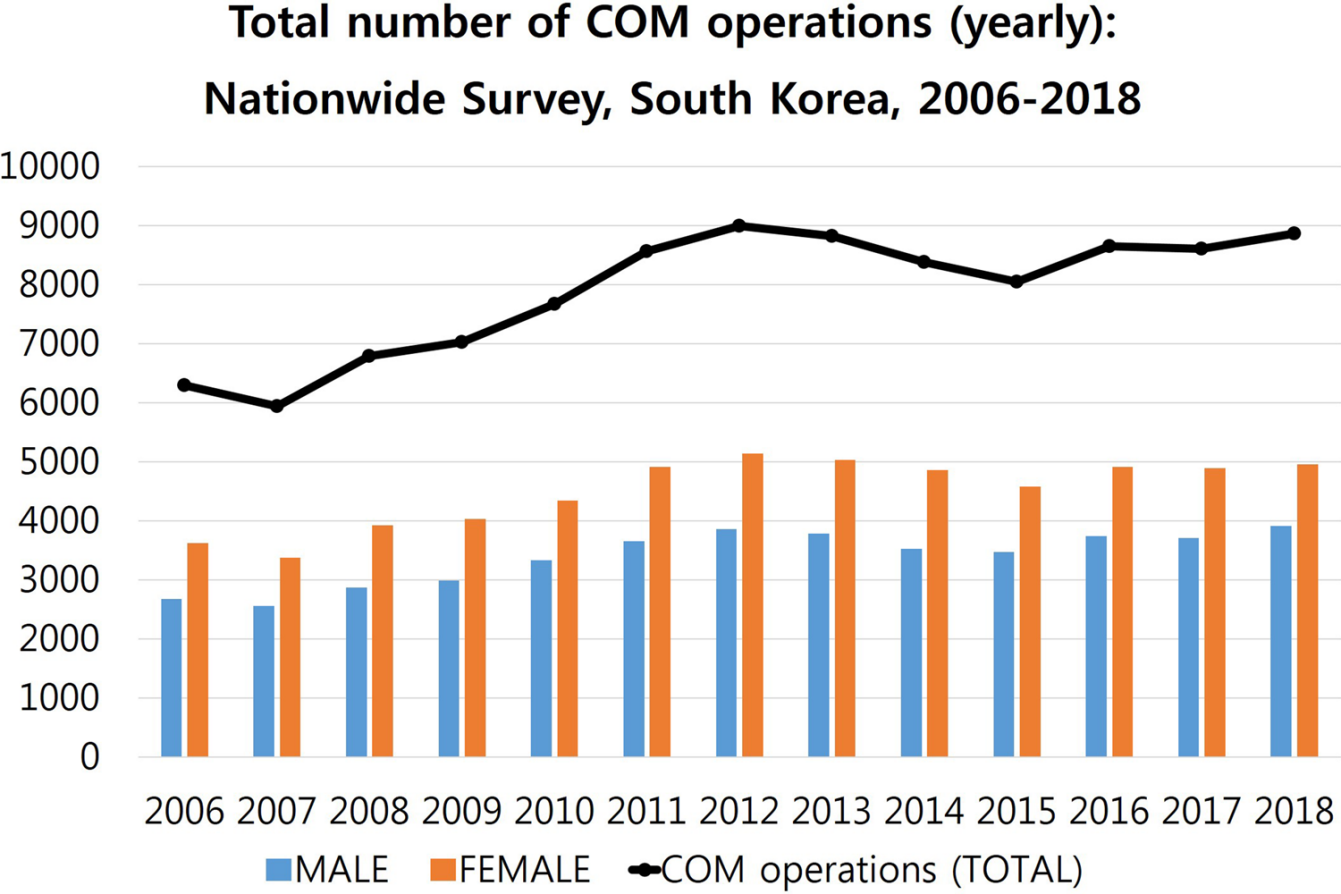
The total number of chronic otitis media (COM) operations in South Korea determined using a nationwide survey to analyse a 13-year trend (2006–2018). The total number of COM operations was lowest in 2007 (5,935 individuals, 0.01205%), highest in 2012 (8,999 individuals, 0.01766%), and formed a plateau until 2018 (8,870 individuals, 0.01711%). The total number of COM operations in females was always higher than that in males. Male/female ratio was slightly increased from 0.738 to 0.790 (2006–2018), which means that more males underwent COM operations recently than before.

The total number of COM operations was lowest in 2007 (5,935 individuals, 0.01205%), highest in 2012 (8,999 individuals, 0.01766%), and formed a plateau until 2018 (8,870 individuals, 0.01711%). The total number of COM operations in females was higher than that in males throughout. The M/F ratio was slightly increased from 0.738 to 0.790 (2006–2018), which means that more males underwent COM operations recently than before (Fig. 2).

### The total number of cholesteatoma surgeries in South Korea (2006–2018)

**Figure 3:**
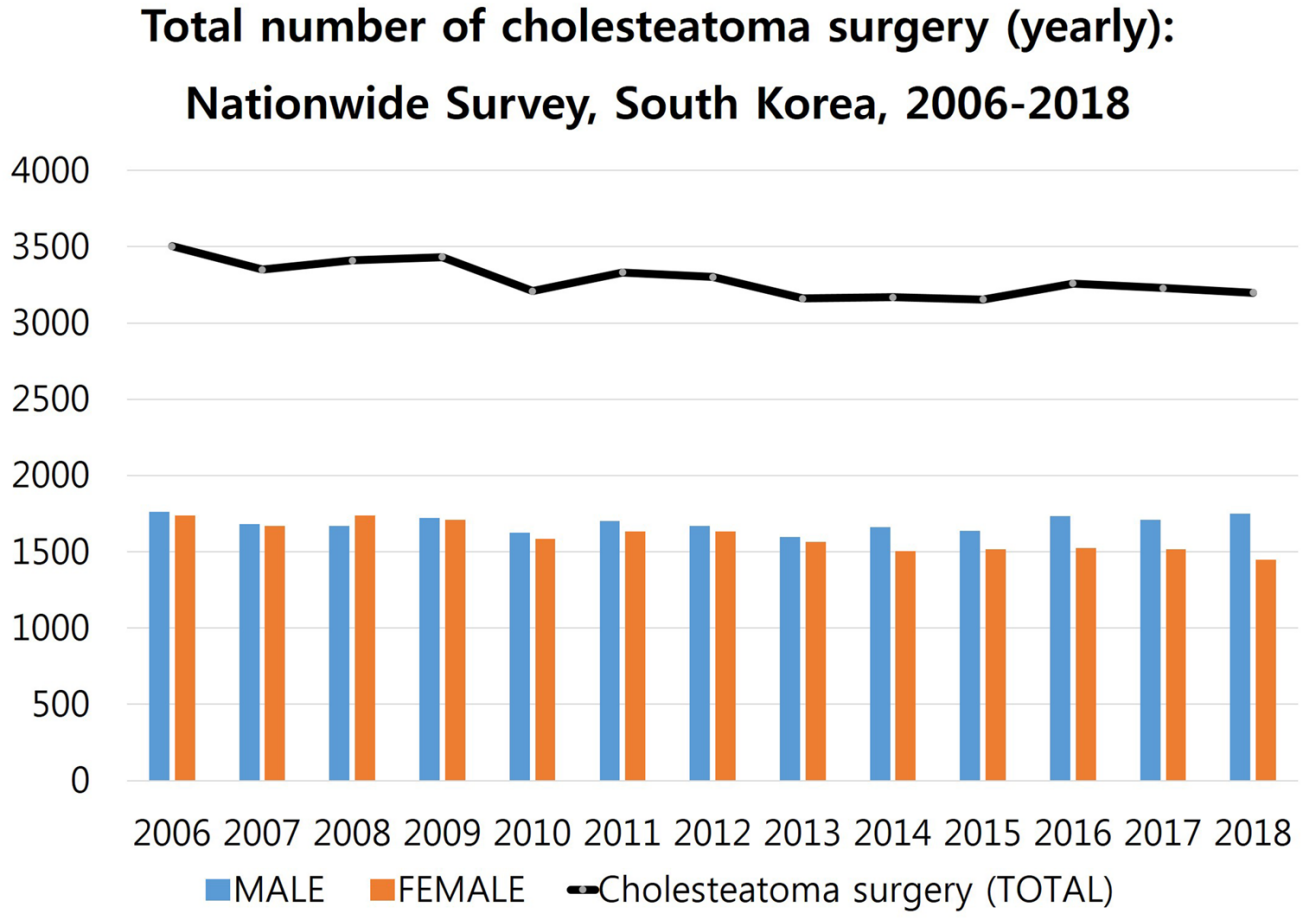
The total number of cholesteatoma surgeries in South Korea determined using a nationwide survey to analyse a 13-year trend (2006–2018). The number was the highest in 2006 (3,502 individuals, 0.00715%), lowest in 2018 (3,199 individuals, 0.00617%), and plateaued until 2018. The rate of cholesteatoma surgery was relatively similar in male and female patients throughout the period. Recently however, cholesteatoma surgery rates were higher (1.2 times higher) in the male population than in the females in 2018.

The total number of cholesteatoma surgeries was highest in 2006 (3,502 individuals, 0.00715%), lowest in 2018 (3,199 individuals, 0.00617%), and decreased gradually until 2018 (Fig. 3). The rate of cholesteatoma surgery was relatively similar in male and female patients throughout the period. Recently, however, cholesteatoma surgery rates were higher in the male population (1.2 times higher than in females in 2018).

### Rate of COM operations and cholesteatoma surgeries in South Korea (2006–2018)

**Figure 4:**
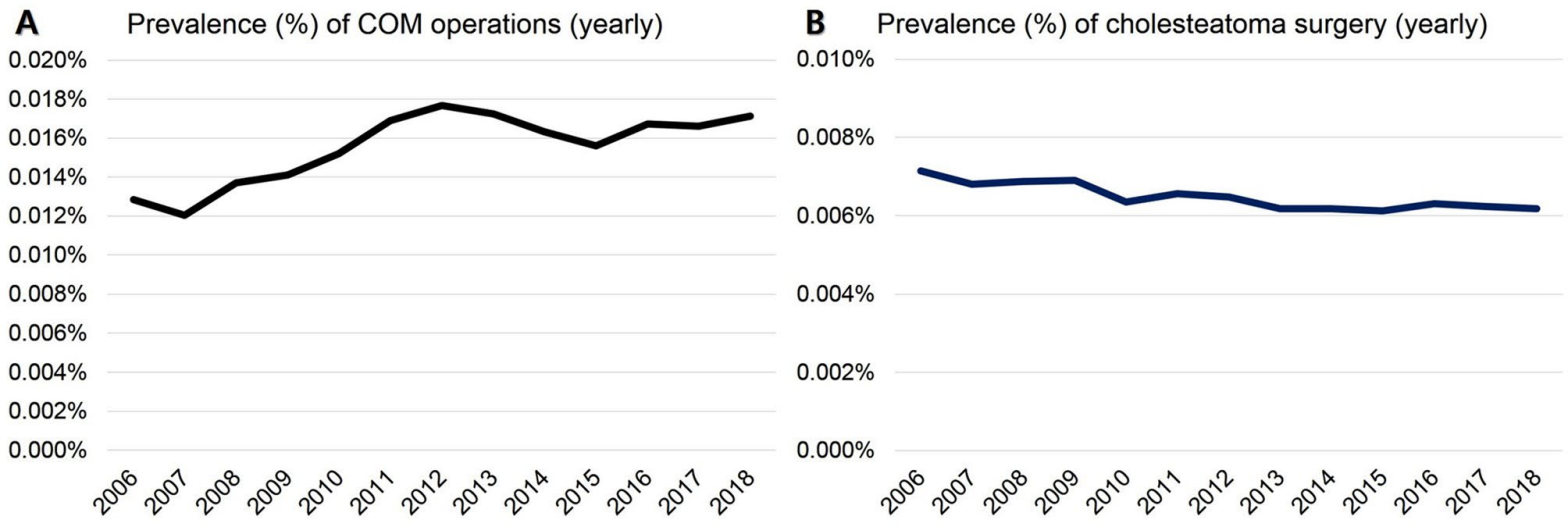
Rates (%) of chronic otitis media (COM) operations and cholesteatoma surgeries in South Korea determined using a nationwide survey to analyse a 13-year trend (2006–2018). Each graphical trend of both total population number and prevalence, were almost same in both COM operations and cholesteatoma surgeries. (**A**) Rates of COM operations were lowest in 2007 (5,935 individuals, 0.01205%), highest in 2012 (8,999 individuals, 0.01766%), and plateaued until 2018 (8,870 individuals, 0.01711%). In 2006, COM operations were 1.8 times more numerous than cholesteatoma surgeries (0.01285 vs. 0.00715%). In 2018, COM operations were 2.8 times more numerous than cholesteatoma surgeries (0.01711 vs. 0.00617%). (**B**) Rates of cholesteatoma surgeries were highest in 2006 (3,502 individuals, 0.00715%), lowest in 2015 (3,154 individuals, 0.00612%), and decreased slowly from 2006 to 2018 (0.00715–0.00617%).

The rate of COM operations was lowest in 2007 (5,935 individuals, 0.01205%), highest in 2012 (8,999 individuals, 0.01766%), and plateaued until 2018 (8,870 individuals, 0.01711%) (Fig. 4A). In 2006, COM operations were 1.8 times more numerous than cholesteatoma surgeries (0.01285% vs. 0.00715%). In 2018, COM operations were 2.8 times more numerous than cholesteatoma surgeries (0.01711% vs. 0.00617%). The total number and rate of COM operations were always higher than those of cholesteatoma surgeries. The rate of cholesteatoma surgery was highest in 2006 (3,502 individuals, 0.00715%), lowest in 2015 (3,154 individuals, 0.00612%), and generally decreased from 2006 to 2018 (0.00715–0.00617%) (Fig. 4B). Each graphical trend of both total population number and rate, was almost the same in both COM operations and cholesteatoma surgeries (Figs. 2, 3 and 4). As time went by, the total number and rate of cholesteatoma surgery decreased from 2006 to 2018.

### The total number of COM operations in South Korea according to age groups (2006–2018)

**Figure 5:**
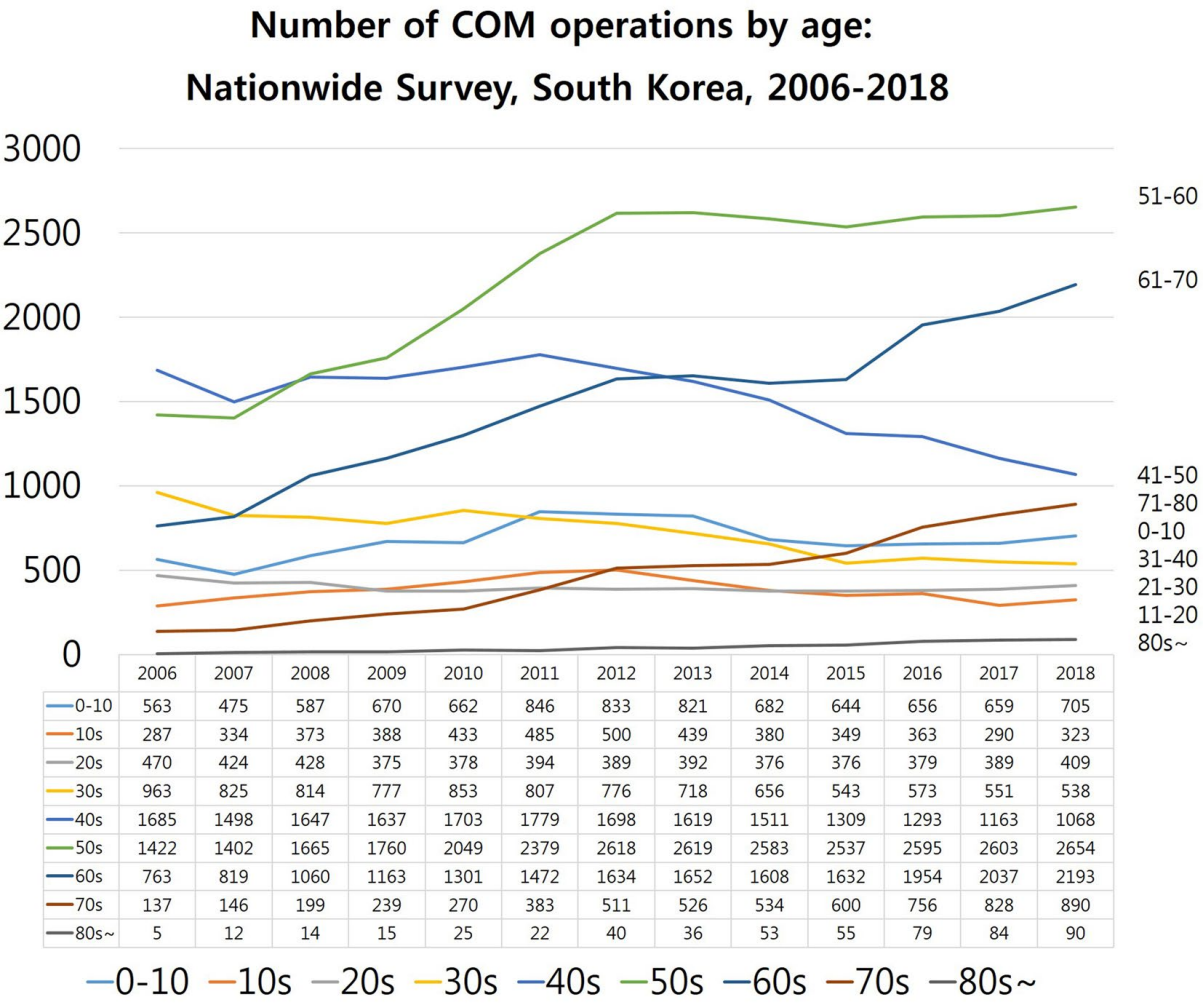
The total number of chronic otitis media (COM) operations in South Korea determined using a nationwide population-based study to analyse a 13-year trend (2006–2018) according to age groups. In 2006, patients in their 40s (41–50 years) represented the predominant age group for COM operations, but the total number of patients in their 40s dramatically decreased from 1685 to 1,068 (from 2006 to 2018 years). The 50s age group which was ranked 2nd in 2006, dramatically increased by 2012, and plateaued after occupying 1st rank. The 60s age group which occupied 4th rank in 2006, dramatically rose to 2nd rank in 2018 because of the ageing population of South Korea. From 2006 to 2018, there was a visible increase in the number of COM operations in patients in their 50s, 60s, and 70s (1st, 2nd, 4th rank in 2018, respectively) due to the ageing population phenomenon. In contrast, there was a decrease in the number of COM operations in patients in their 40s and 30s (3rd, 6th rank in 2018, respectively).

In 2006, patients in their 40s (41–50 years) represented the predominant age group for COM operations, but the total number of patients in their 40s dramatically decreased from 1685 to 1,068 (from 2006 to 2018 years). The 50s age group which was ranked 2nd in 2006 dramatically increased till 2012, and plateaued while ranking 1st (Fig. 5). The 60s age group ranked 4th in 2006 and dramatically increased to the 2nd rank in 2018 due to ageing population in South Korea. From 2006 to 2018, there was a prominent increase in the number of COM operations in patients in their 50s, 60s, and 70s (1st, 2nd, 4th rank in 2018, respectively) due to senescence in the population. In contrast, there was a decrease in the number of COM operations in those in their 40s and 30s (3rd and 6th rank in 2018, respectively).

### The total number of cholesteatoma surgeries in South Korea according to age groups (2006–2018)

**Figure 6:**
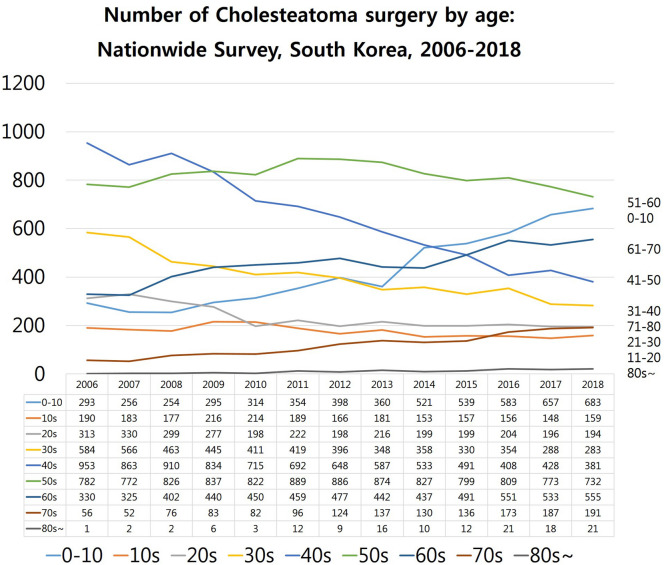
The total number of cholesteatoma surgeries in South Korea determined using a nationwide population-based study to analyse a 13-year trend (2006–2018) according to age groups. According to the 2018 data, cholesteatoma surgeries were most commonly performed in patients in their 50s. The 50s age group was ranked 2nd in 2006, plateaued by 2018 after rising to 1st rank. Cholesteatoma surgery rates increased dramatically from 2006 to 2018 in patients aged 0–10 years due to congenital cholesteatoma (6th rank in 2006 to 2nd rank in 2018). Cholesteatoma surgery rates also increased in patients aged 61–80 years due to senescence in the population. The 60s age group which was ranked 4th in 2006, gradually rose to 3rd rank in 2018. Cholesteatoma surgery rates decreased in patients aged 41–50 years, from ranking 1st in 2006 to 4th in 2018 (from 953 to 381 individuals). From 2006 to 2018, the 50s and 60s (1st, 3rd rank in 2018, respectively) were the prominent age groups due to acquired cholesteatoma. In contrast, the 0–10 years age group rose from 6th rank in 2006 to 2nd rank in 2018, mainly due to congenital cholesteatoma.

According to the 2018 data, cholesteatoma surgery was most commonly performed in patients in their 50s (Fig. 6). The 50s age group occupied 2nd rank in 2006 and plateaued till 2018 after ascending to the 1st rank. Cholesteatoma surgery rates increased dramatically from 2006 to 2018 in patients aged 0–10 years due to congenital cholesteatoma (6th rank in 2006 to 2nd rank in 2018). Cholesteatoma surgery rates also increased in patients aged 61–80 years due to ageing population. The 60s age group which ranked 4th in 2006 gradually rose to the 3rd rank in 2018. Cholesteatoma surgery rates decreased in patients aged 41–50 years, ranking 1st in 2006 and falling to the 4th rank in 2018 (from 953 to 381 individuals). From 2006 to 2018, the prominent age groups were the 50s and 60s (1st, 3rd rank in 2018, respectively) due to acquired cholesteatoma. In contrast, there was a prominent rise of the 0–10 age group from 6th rank in 2006 to 2nd rank in 2018, due to congenital cholesteatoma (Fig. 6).

### Summary of chronic otitis media operations and cholesteatoma surgeries in South Korea using a nationwide survey to analyse a 13-year trend (2006–2018)

In each COM operation and cholesteatoma surgery group, male/female classification datasets were evaluated, and analyses using age-related grading were acquired. COM operations increased from 2006 to 2018, and the graphical trend was similar to that of the total population of South Korea. However, cholesteatoma surgery decreased from 2006 to 2018 in both the total number and the rate. For COM operations, the total number of females was always higher than males, and the M/F ratio was increased from 0.738 to 0.79. For cholesteatoma surgery, the total number of males was higher than females, and the M/F ratio was increased from 1.014 to 1.206 (Table 1).

**Table 1:** The total number of chronic otitis media (COM) operations and cholesteatoma surgeries in South Korea determined using a nationwide survey to analyse a 13-year trend (2006–2018). In each COM operation and cholesteatoma surgery group, male/female classification datasets were evaluated, and the analyses using age-related grading were acquired. COM operations increased from 2006 to 2018, and the graphical trend was like that of the total population of South Korea. However, cholesteatoma surgeries decreased from 2006 to 2018 in both total number and rate. For COM operations, the total number of female was always higher than males, and the M/F ratio was increased from 0.738 to 0.79. For cholesteatoma surgery, the total number of males was higher than females, and the M/F ratio was increased from 1.014 to 1.206.

	2006	2007	2008	2009	2010	2011	2012	2013	2014	2015	2016	2017	2018
**Chronic otitis media (COM) operations**	6,295	5,935	6,787	7,024	7,674	8,567	8,999	8,822	8,383	8,045	8,648	8,604	8,870
Percentage	0.01285	0.01205	0.01370	0.01411	0.01519	0.01689	0.01766	0.01725	0.01633	0.01561	0.01673	0.01662	0.01711
**Sex**													
Male	2,674	2,558	2,865	2,992	3,330	3,654	3,859	3,787	3,527	3,471	3,740	3,711	3,915
Female	3,621	3,377	3,922	4,032	4,344	4,913	5,140	5,035	4,856	4,574	4,908	4,893	4,955
M/F ratio	0.738	0.757	0.730	0.742	0.767	0.744	0.751	0.752	0.726	0.759	0.762	0.758	0.790
**AGE grading**													
0–10	563	475	587	670	662	846	833	821	682	644	656	659	705
11–20	287	334	373	388	433	485	500	439	380	349	363	290	323
21–30	470	424	428	375	378	394	389	392	376	376	379	389	409
31–40	963	825	814	777	853	807	776	718	656	543	573	551	538
41–50	1,685	1,498	1,647	1,637	1,703	1,779	1,698	1,619	1,511	1,309	1,293	1,163	1,068
51–60	1,422	1,402	1665	1,760	2049	2,379	2,618	2,619	2,583	2,537	2,595	2,603	2,654
61–70	763	819	1,060	1,163	1,301	1,472	1,634	1,652	1,608	1,632	1,954	2,037	2,193
71–80	137	146	199	239	270	383	511	526	534	600	756	828	890
81–	5	12	14	15	25	22	40	36	53	55	79	84	90

	2006	2007	2008	2009	2010	2011	2012	2013	2014	2015	2016	2017	2018
**Cholesteatoma surgeries**	3,502	3,349	3,409	3,433	3,209	3,332	3,302	3,161	3,168	3,154	3,259	3,228	3,199
Percentage	0.00715	0.00680	0.00688	0.00690	0.00635	0.00657	0.00648	0.00618	0.00617	0.00612	0.00630	0.00623	0.00617
**Sex**													
Male	1,763	1,681	1,669	1,723	1,624	1,700	1,669	1,598	1,663	1,637	1,736	1,711	1,749
Female	1,739	1,668	1,740	1,710	1,585	1,632	1,633	1,563	1,505	1,517	1,523	1,517	1,450
M/F ratio	1.014	1.008	0.959	1.008	1.025	1.042	1.022	1.022	1.105	1.079	1.140	1.128	1.206
**AGE grading**													
0–10	293	256	254	295	314	354	398	360	521	539	583	657	683
11–20	190	183	177	216	214	189	166	181	153	157	156	148	159
21–30	313	330	299	277	198	222	198	216	199	199	204	196	194
31–40	584	566	463	445	411	419	396	348	358	330	354	288	283
41–50	953	863	910	834	715	692	648	587	533	491	408	428	381
51–60	782	772	826	837	822	889	886	874	827	799	809	773	732
61–70	330	325	402	440	450	459	477	442	437	491	551	533	555
71–80	56	52	76	83	82	96	124	137	130	136	173	187	191
81 ~	1	2	2	6	3	12	9	16	10	12	21	18	21

## Discussion

This study is the first official big data analysis on otologic surgery in South Korea conducted using a nationwide database—the NHID operated by the KNHIS and conducted by the Korean Audiological Society. This study surveyed the entire population of over 50 million in South Korea, and not just a representative population or a part of the national population.

South Korea has advanced from being a developing country and has joined the ranks of advanced countries. The economy of South Korea has been growing ($1.012 trillion in 2006 to $1.720 trillion in 2018; ranked 12th in the world), and GNI per capita has increased from $19,950 in 2006 to $30,600 in 2018, 28th world rank in 2018. Accordingly, medical services and disease prevention have also been improving; however, the South Korean population is rapidly ageing into “ageing society”. Therefore, the analysis of trends of otologic surgery according to year, sex, and age is very important and valuable in this turbulent period.

As far as we know, there have been few reports of nationwide big data analysis of otologic surgery. It can be predicted that the number and trend of surgeries can be very different because COM operations or cholesteatoma surgeries are influenced by various factors such as COM prevalence, medical condition, medical accessibility, environment, and life expectancy of each country.

In previous studies, the prevalence of HL is usually decreased when a country becomes an advanced country, even though the population tends to become an ageing one^14^. As such, the total number of COM operations had peaked in 2012 and was also stabilised until 2018 (0.01711%) in South Korea. In the case of cholesteatoma surgeries, the total number decreased slowly from 2006 to 2018 (0.00715–0.00617%). These observations can be confirmed by showing the same result in the rate (%) as well as the total number of COM operations and cholesteatoma surgeries (Fig. 4). In this study, the number of COM operations in the 50s and 60s age groups increased because of the ageing society. In contrast, acquired-type cholesteatoma was prominent in the 50s age group, and congenital cholesteatoma was prominent in the age group of 0–10 years. In addition, the proportion of women undergoing COM operations was higher, whereas the proportion of men was higher for cholesteatoma surgery.

As countries became advanced and medical care developed, COM operation rates decreased^1,3,14^. Thus, in this study, the total number/rate of COM operation was decreased too. The total number/rate of cholesteatoma surgery decreased as time went by, even though the total population of South Korea continued to increase. The most important reason for cholesteatoma’s decrease could be the popular use of ventilation tubes (VT). There is a report of significant decrease in the rates of cholesteatoma surgeries when comparing the 10 years (1961–1970; 413 cases) before VT use in secretory otitis media to the last 10 years (1989–1998; 228 cases) following the use of VT^17^. In addition, better hygiene and complete vaccination can be positive factors to control inflammation of ear disease, and the development of MRI sequences in the early 2000s can help facilitate the detection of primary and residual lesions^17^. Interestingly, the proportion of women is higher in COM operations, and several studies have reported similar results^6,7^. It is presumed that chronic otitis media or wet ear is more likely to develop because women's ear canals are smaller than men’s.

Considering the total population data of South Korea by age (Figure S1), age groups in order to number of population is as follows: 40s–50s–30s–20s–10/60s–0–9–70s–80s. The number of COM operations in the 50s and 60s age groups was significant, the reasons being (1) the ageing society, (2) symptoms of COM such as otorrhea may be aggravated as immunity or infection control decreased with age, (3) less anaesthesia and general surgery risks. The number of COM operations was small in the 80s age group because of higher surgical risks such as anaesthesia and healing problems. Considering the rate of COM operation by age, the rate was about 3% in 50s, about 3.7% in 60s, and the rate of COM operation was stable recently.

Interestingly, the generation which received COM surgery mostly, could be same as the 40s in 2006 and the 50s in 2018. National Health Insurance started in 1977 with large corporations, and was completed in 1987 as KNHIS for all citizens in Korea. After that, proper treatment of ear diseases has become popular in South Korea, and such treatments include VT insertion, vaccination, better hygiene, adequate antibiotics, and COM surgery. Thus, there can be a strong inflection point with reduced COM surgery or ear disease, which point may be a national insurance service for all people.

Acquired cholesteatoma was prevalent in patients in their 50s and 40s, but decreased from 2006 to 2018. Congenital cholesteatoma was prevalent and increased in the 0–10 years age group, although total cholesteatoma decreased from 2006 to 2018. Considering the rate of cholesteatoma surgery by age, the rate was about 1.5% in the 0–10 years age group, and the rate of cholesteatoma surgery also was increasing in the 0–10 group recently. The development of ENT endoscopic systems can be the major reason for early detection of congenital cholesteatoma.

Table 2 describes the annual incidence of COM and cholesteatoma in each nation from the review of literature. The annual incidence of COM was 4.2–17/100,000 individuals, and the annual incidence of cholesteatoma was 5–66/100,000^9–12,18–21^. In this study, the annual rate of COM operations was 17/100,000 individuals, and of cholesteatoma surgeries was 6/100,000 in South Korea.

**Table 2:** Annual incidence of chronic otitis media (COM) operations and cholesteatoma surgeries in each nation from the review of literature. In this study, annual incidence of COM operations was 17/100,000 individuals, and cholesteatoma surgery was 6/100,000 in South Korea.

Annual incidence of surgery	COM	Cholesteatoma	Nation
Ruben	4.2/100,000		US
Homoe and Bretlau		5/100,000	Greenland
Harker		6/100,000	IOWA, US
IM, GJ	17/100,000	6/100,000	South Korea
Britze		6.8/100,000	Denmark
Kemppainen		9.2/100,000	Finland
Shibata		6.8–10/100,000	Japan
Olszenska		12.2/100,000	Poland
Padgham		13/100,000	Scotland
Djurhuus		8–15/100,000	Denmark
TOS		15.6/100,000	Denmark

In conclusion, the annual rate of COM operations was 0.017% in South Korea, and this rate seems to be plateaued/decreased after a peak point in the advanced country. The rate of cholesteatoma surgery was 0.006%, and decreased annually. There was a female predominance in COM operations, but male predominance in cholesteatoma surgeries. Major age groups for COM/Cholesteatoma surgeries were the 50s and 60s, and congenital cholesteatoma of childhood (0–10 years) accounted for about 20% of all cholesteatoma surgeries.

## Limitations

In this study, a possible limitation was that some COM/cholesteatoma patients could not undergo COM/cholesteatoma operations, as such the rate of COM/cholesteatoma operations was underestimated. Early-stage cholesteatoma cases, which require only atticotomy or attic reconstruction, may be not included, and recent endoscopic ear surgery for COM/cholesteatoma may be not included too (no mastoidectomy). Unfortunately, the revision surgery cases were not distinguished from standard COM/cholesteatoma operations but were calculated together.

However, trends of the rates of COM/cholesteatoma operations observed in this study are solid, because the KNHIS covers the entire national population and all patients admitted to hospital for COM/cholesteatoma operations were counted in this study.

### Further recommendations for research

In the future, the KNHIS should be tailored to classify tympanoplasty according to different types, such as type 1–4 for further analysis of otologic operations. Ideal future study includes multivariate analyses of risk factors in otologic surgery and analyses of pre/post-operation hearing results in major otologic surgeries.

## Methods

This study used nationwide big data from NHID, which is operated by the KNHIS, a government-affiliated agency under the Korean Ministry of Health and Welfare that supervises all medical data in South Korea. All Korean citizens and registered foreigners must be enrolled, and receive medical services from the KNHIS. All patients with COM operations and cholesteatoma surgeries are registered in the KNHIS of South Korea. Medical data for patients of all ages were extracted retrospectively from the NHID from January 2006 to December 2018 (NHIS-2018). Retrospective medical data from the NHID do not involve any specific personal data such as name, but provide age, gender, number of patients, and the national classification code of disease. The NHID contains information on patients' demographics, medical service use, medication, deductions, and claims. The KNHIS approved this nationwide study and the requirement for informed consent was waived.

In this study, we analysed the sex, age, and yearly trends of registered patients with COM operations or cholesteatoma surgeries. To investigate the trends in surgery rates over 13 years, we also examined the rates of COM operations or cholesteatoma surgeries according to age, sex, and region. In this study, “COM operation” or “cholesteatoma surgery” were defined as (1) admission to an approved hospital, (2) diagnosed with COM or cholesteatoma which was confirmed by temporal bone CT and pathologic report, (3) underwent ICW operation (S5671 code) or OC operation (S5672 code), (4) underwent tympanoplasty (S5640 code). The patients that underwent only tympanoplasty, only myringoplasty, and only mastoidectomy, were excluded from the study.

This study was conducted by the Research Committee of the Korean Society of Otorhinolaryngology-Head and Neck Surgery (http://www.korl.or.kr), and the Korean Audiological Society (http://www.audiosoc.or.kr) reviewed and confirmed the study. As a representative hospital, the institutional review board of the Korea University College of Medicine approved this study (KUMC IRB 2018AN0341). All methods were employed in accordance with the relevant guidelines and regulations.

Statistical data analysis was performed using Microsoft Excel software version 2016 (Microsoft, Redmond, Washington, USA). This nationwide study used actual data from the entire population of South Korea, and hence, no data on standard deviation were available, and there was little need to perform an analysis for predicting trends in a universal population. All the data generated or analysed during this study are available from the corresponding author on reasonable request.

## Supplementary information

Supplementary file1
