# Supplementary material for: Rate of chronic otitis media operations and cholesteatoma surgeries in South Korea: a nationwide population-based study (2006–2018)
Source: Sci Rep. 2020 Jul 9;10:11356. doi: 10.1038/s41598-020-67799-5 (PMC7347575; doi:10.1038/s41598-020-67799-5)

**Supplementary Information**

**Title: Rate of chronic otitis media operations and cholesteatoma surgeries in South Korea: a nationwide population-based study (2006**–**2018)**

**Gi Jung Im^1*^, Kyung do Han^2^, Kyung Ho Park^3^, Chang Hyun Cho^4^, Hyunsook Jang^5^, Jun Ho Lee^6^, Seung Hwan Lee^7^***

^1^Department of Otolaryngology-Head and Neck Surgery, Korea University College of Medicine

^2^Department of Statistics and Actuarial Science, Soongsil University

^3^Department of Otolaryngology-Head and Neck Surgery, Catholic University College of Medicine

^4^Department of Otolaryngology-Head and Neck Surgery, Gachon University College of Medicine

^5^Division of Speech Pathology and Audiology, Hallym University College of Natural Sciences

^6^Department of Otolaryngology-Head and Neck Surgery, Seoul University College of Medicine

^7^Department of Otolaryngology-Head and Neck Surgery, Hanyang University College of Medicine, Seoul, Korea

[*logopas@korea.ac.kr](mailto:*logopas@korea.ac.kr); earkorea@gmail.com

**Figure S1.** Total population of South Korea by AGE from 2008 to 2017. Major population of South Korea is 40s. Population of 50s, 60s, 70s, and 80s, increases gradually from 2008 to 2017, which means “ageing population” of South Korea. Youth population shows a decreasing trend.


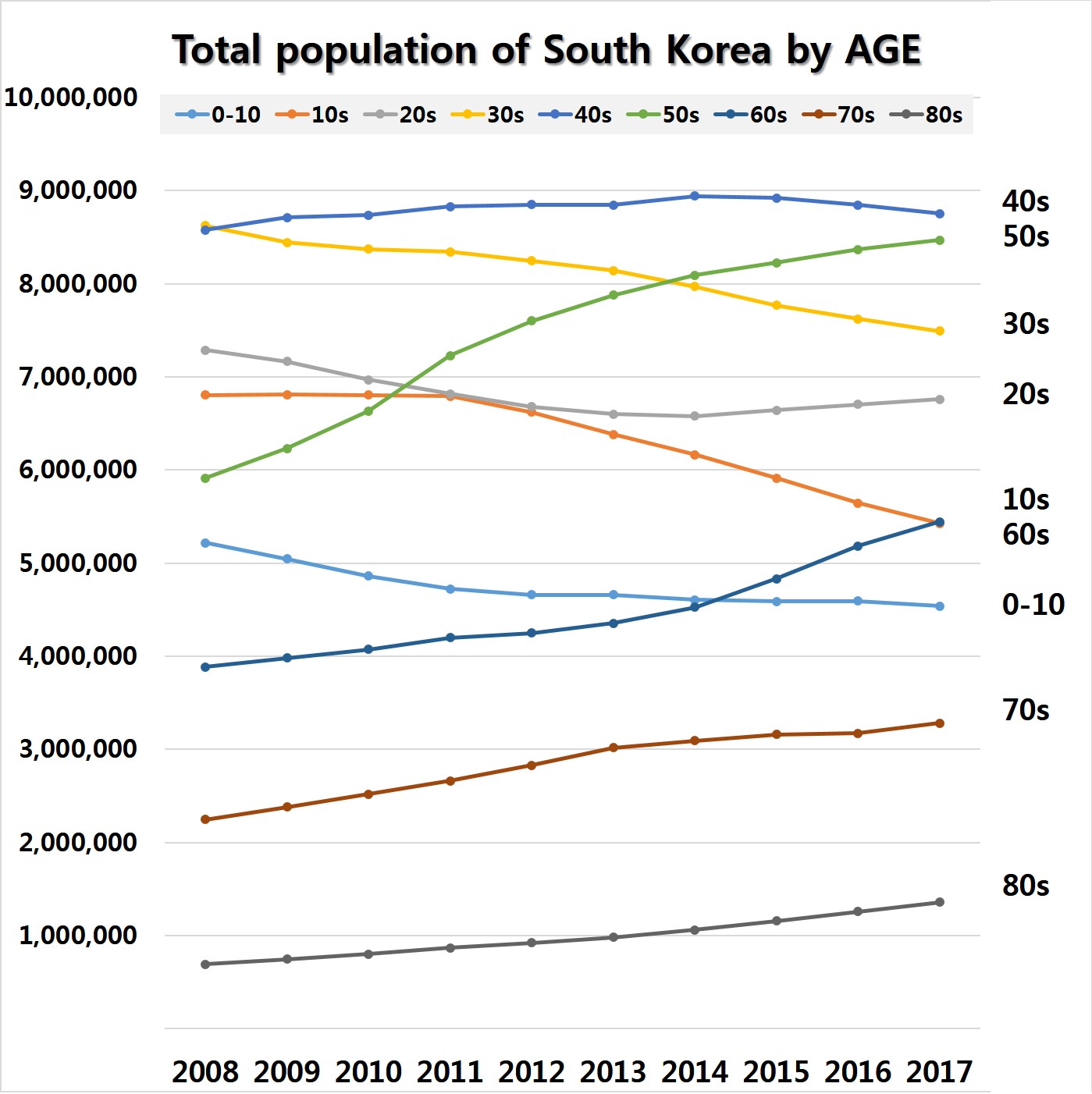

Supplement: Supplementary file 1 — Supplementary file1 [file 41598_2020_67799_MOESM1_ESM.docx]
